# Computer simulations reveal complex distribution of haemodynamic forces in a mouse retina model of angiogenesis

**DOI:** 10.1098/rsif.2014.0543

**Published:** 2014-10-06

**Authors:** Miguel O. Bernabeu, Martin L. Jones, Jens H. Nielsen, Timm Krüger, Rupert W. Nash, Derek Groen, Sebastian Schmieschek, James Hetherington, Holger Gerhardt, Claudio A. Franco, Peter V. Coveney

**Affiliations:** 1 CoMPLEX, University College London, Physics Building, Gower St., London WC1E 6BT, UK; 2 Centre for Computational Science, Department of Chemistry, University College London, 20 Gordon St., London WC1H 0AJ, UK; 3 Vascular Biology Laboratory, London Research Institute, Cancer Research UK, Lincoln's Inn Laboratories, 44 Lincoln's Inn Fields, London WC2A 3LY, UK; 4 Research Software Development Team, Research Computing and Facilitating Services, University College London, Podium Building—1st Floor, Gower St., London WC1E 6BT, UK; 5 Institute for Materials and Processes, School of Engineering, University of Edinburgh, King's Buildings, Mayfield Road, Edinburgh EH9 3JL, UK; 6 Instituto de Medicina Molecular, Faculdade de Medicina, Universidade de Lisboa, Lisboa 1649-028, Portugal

**Keywords:** angiogenesis, mouse, retina, blood flow, shear stress, lattice-Boltzmann

## Abstract

There is currently limited understanding of the role played by haemodynamic forces on the processes governing vascular development. One of many obstacles to be overcome is being able to measure those forces, at the required resolution level, on vessels only a few micrometres thick. In this paper, we present an *in silico* method for the computation of the haemodynamic forces experienced by murine retinal vasculature (a widely used vascular development animal model) beyond what is measurable experimentally. Our results show that it is possible to reconstruct high-resolution three-dimensional geometrical models directly from samples of retinal vasculature and that the lattice-Boltzmann algorithm can be used to obtain accurate estimates of the haemodynamics in these domains. We generate flow models from samples obtained at postnatal days (P) 5 and 6. Our simulations show important differences between the flow patterns recovered in both cases, including observations of regression occurring in areas where wall shear stress (WSS) gradients exist. We propose two possible mechanisms to account for the observed increase in velocity and WSS between P5 and P6: (i) the measured reduction in typical vessel diameter between both time points and (ii) the reduction in network density triggered by the pruning process. The methodology developed herein is applicable to other biomedical domains where microvasculature can be imaged but experimental flow measurements are unavailable or difficult to obtain.

## 1. Introduction

Despite recent advances in vascular biology, the mechanisms underpinning vascular development remain poorly understood. It is therefore crucial to gain further insight into the mechanisms governing the formation of complex vascular networks and their response to external stimuli. The translation of these results holds the key to the improvement of therapies modulating vascular patterning and sprouting for the treatment of stroke, ischaemia, retinopathies or cancer, the leading cause of death worldwide.

**Figure 1.**
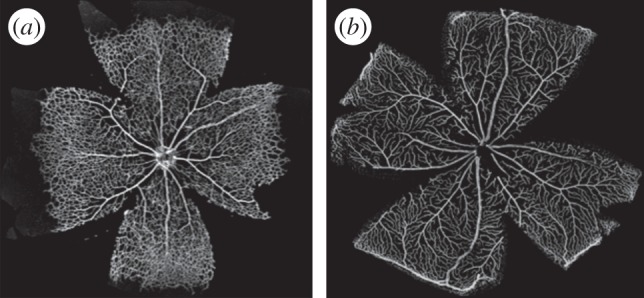
Murine retinal vascular plexus 6 and 21 days postnatal (panel (*a*) and (*b*), respectively). Within days, the primitive vessel network remodels into mature vasculature. Samples were collected, mounted and imaged as described in §3.

One of the pressing questions in the field is establishing how primitive vessel networks remodel into a hierarchically branched and functionally perfused network of arteries, arterioles, capillaries and venules (figure 1). In recent years, the main molecular mechanisms regulating endothelial cell behaviour during vessel formation have been elucidated using experimental techniques [1,2]. However, important challenges remain: (i) understanding how cell-level mechanisms integrate to give rise to systems-level behaviour and (ii) understanding the impact in vascular patterning of the interplay between cellular molecular regulation and haemodynamic forces (i.e. vascular mechanotransduction). These problems are hard to address due to the multiscale and multiphysics nature of the processes involved. Systems-level behaviour arises from highly nonlinear, tightly coupled interactions between subprocesses at different spatial and temporal scales. Furthermore, it has been recently proposed [3] that a tighter integration between experimental and computational work is required in order to tackle these questions. Working in a feedback loop, computational models should be capable of generating new hypotheses, rather than merely reproducing experimental data. In turn, experiments should provide new biological insights based on these hypotheses and help to further refine computational models.

Multiple animal models have been proposed for the study of vascular development. Examples include the mouse retinal and embryonic vasculature [4], zebrafish vasculature [5,6] and hyaloid vasculature [7]. In recent years, there has been increasing interest in the development of *in silico* models for the close inspection of certain vascular developmental aspects. To date, most work concerning simulation of retinal haemodynamics for the study of vascular mechanotransduction (see §2.3 for a review) has suffered from a number of limitations including: (i) limited availability of spatial information due to the use of low-resolution imaging modalities, (ii) oversimplification of the haemodynamics by considering the retinal plexus to be a network of one-dimensional vessel segments and (iii) unavailability of the computer code developed. We believe that the model simplifications cited, although appropriate in some applications, may fail to capture complex flow patterns important for understanding the interplay between molecular regulation and haemodynamics during development. Hence, in this work, we introduce a computational workflow—and make the source code available—aimed at generating *in silico* estimates of the haemodynamic forces acting on samples of mouse retinal vasculature imaged during development, typically within the first postnatal week. The workflow involves the following steps. First, high-resolution scanning confocal microscope images are obtained and segmented in order to generate a binary mask of the vessel lumen. Second, luminal centrelines and radii are computed in a process known as skeletonization. Next, three-dimensional models of the luminal surface are reconstructed based on the computed skeleton. Finally, blood flow simulations are run in order to obtain estimates of blood velocity and wall shear stress (WSS) with an open-source highly parallel computational fluid dynamics (CFD) solver, known as HemeLB [8].

The purpose of this paper is therefore threefold. First, to describe the computational methods developed and to survey the literature for data not accessible in our experiments but necessary for model set-up. Second, to validate our methods in simplified scenarios where analytical solutions are known. Third, to present and to analyse simulations in order to gain insight into the dynamics of retinal blood flow during development. The paper is structured as follows. In §2, we survey the literature for previously proposed models of retinal flow and for the experimental data necessary to set up our simulations. Next, in §3, we present the methods used for image processing and three-dimensional model reconstruction as well as the validation methodology adopted. Section 4 presents the main results on model reconstruction, validation and a set of simulations on the reconstructed three-dimensional models. Finally, §5 summarizes the main contributions of the work and outlines the areas where we plan to apply the computational pipeline developed.

## 2. Retinal vascular structure and flow

### 2.1. Vascular structure and its development

Angiogenesis defines the formation of new blood vessels from pre-existing ones and can be split into two distinct phases: sprouting and remodelling. During sprouting, new vessels form and invade avascular ischaemic areas, where tissues experience hypoxia and nutrient deprivation. This process is modulated by the secretion of various growth factors, including vascular endothelial growth factor (VEGF), through a cascade of signalling events. The endpoint of this phase is the formation of a highly branched and poorly perfused network of capillary connections. Remodelling is responsible for the creation of a hierarchically branched and efficient vascular tree, containing defined arteries and veins and an optimized vascular capillary network. A vital step during vascular remodelling is the removal of redundant vessel segments: vessel pruning. Importantly, angiogenesis is a very dynamic process occurring not only during development but also in adulthood (e.g. wound healing and tumour formation).

The neonatal mouse retina has become one of the main experimental models for the study of the mechanisms involved in blood vessel development and patterning [9–11]. The mouse retina is avascular at birth and develops through a consistent series of events. Astrocytice (a type of glial cell) and neuron-derived vascular endothelial growth factor A (VEGFA) stimulates sprouting angiogenesis from pre-existing blood vessels at the optic nerve. Under a gradient of VEGFA, vessels expand radially in the superficial layer of the retina, in a very characteristic pattern [2]. It takes this process around 8 days to cover the entire surface of the mouse retina. Vascularization of the superficial layer is followed by a second phase of sprouting, where endothelial cells from the superficial venous plexus sprout and penetrate the deeper layers of retina to form, firstly, a deep and, secondly, an intermediate capillary bed [12]. The vascular plexus finally matures about 20 days after birth.

In the mature mouse retina, vessels are found to be predominantly arteriolar in the superficial layer and predominantly venular in the deep capillary bed [13]. The artery feeding the retina arrives at the optic disc and divides into eight to nine radiating retinal arteries. These arteries, with luminal diameter of up to 28 μm [14], side branch into smaller arterioles at close to 90° angles from the parent arteries. The arterioles (10–12 μm in diameter [14]) take a relative long course before abruptly changing direction to run towards the intermediate and subsequently deep capillary beds (5–6 μm in diameter [14]). Some authors (e.g. Paques [13]) have suggested that the superficial layer is mostly capillary-free with only a few direct connections between arteries and veins.

### 2.2. Haemodynamics

The relationship between haemodynamics and pathogenesis of various eye disorders has prompted researchers to analyse retinal blood flow in both basic and clinical research domains. Early examples are the work of Feke *et al.* [15], who measured total retinal blood flow and its regional distribution in humans, and Alm & Bill [16] who studied blood flow rates in various tissues of the primate eye. Later advances in imaging techniques (e.g. optical coherence tomography and related modalities, laser Doppler velocimetry) have expanded our understanding of retinal haemodynamics. High-resolution *in vivo* measurements of retinal flow have been obtained in various species: mouse [12], rat [17] and human [18,19]. Several authors [18,20] presented evidence of the pulsatile nature of retinal blood flow despite early claims [16] that only retinal arteries—and not veins—exhibit systolic to diastolic flow rate variations. Further developments enabled quantitative analysis of typical arterial and venous flow in both healthy [21] and diseased [19] human retinas as well as during development in mice [12]. Table 1 compiles some of the measurements of murine and human retinal vessel diameter, velocity and flow rate available in the literature.

**Table 1.** Experimentally observed values of vessel diameter and flow rate in different parts of the murine and human adult retina as reported in various publications. BTBR and C57BL/6J are two common mouse strains used as models of human disease. Values given as mean ± s.e. of the mean, when possible. The values reported by Zhi *et al.* [22] are measured at five different arteries/veins and averaged over three independent measurements. Significant inconsistencies are found across the surveyed literature: (a) Wright *et al.* [23] and Wang *et al.* [21] measured flow rates approximately one order of magnitude higher than Zhi *et al.* [22] and (b) the arterial diameter measured in Ninomiya & Inomata [14] is substantially lower than in Wright *et al.* [23].

authors	species	measurement	value
Wright *et al.* [23]	30-week-old C57BL/6J male mice	arterial diameter	∼57 μm
		venous diameter	∼62 μm
		mean arterial velocity	∼25 mm s^−1^
		mean venous velocity	∼24 mm s^−1^
		arterial flow rate	∼3.9 μl min^−1^
		venous flow rate	∼4.8 μl min^−1^
Zhi *et al.* [22]	22-week-old BTBR female mice	arterial flow rate	(0.40 ± 0.04) μl min^−1^
			(0.55 ± 0.06) μl min^−1^
			(0.50 ± 0.05) μl min^−1^
			(0.48 ± 0.05) μl min^−1^
			(0.40 ± 0.04) μl min^−1^
			(0.45 ± 0.06) μl min^−1^
		venous flow rate	(0.45±0.04) μl min^−1^
			(0.62 ± 0.06) μl min^−1^
			(0.60 ± 0.04) μl min^−1^
			(0.59 ± 0.05) μl min^−1^
			(0.38 ± 0.03) μl min^−1^
			(0.63 ± 0.06) μl min^−1^
Wright *et al.* [24]	11–12-week-old C57BL/6 male mice	arterial diameter	(60.4 ± 0.7) μm
		venous diameter	(69.3 ± 1.3) μm
		mean arterial velocity	(28.3 ± 1.4) mm s^−1^
		mean venous velocity	(26.3 ± 1.2) mm s^−1^
Ninomiya & Inomata [14]	4-month-old mice (*ex vivo*)	arterial diameter	up to 28 μm
		capillary diameter	5 μm to 6 μm
Wang *et al.* [21]	adult human	arterial diameter	(91.23 ± 11.80) μm
		arterial peak velocity	(24.15 ± 1.50) mm s^−1^
		arterial flow rate	(6.83 ± 1.75) μl min^−1^
		venous diameter	(69.83 ± 3.52) μm
		venous peak velocity	(46.43 ± 1.42) mm s^−1^
		venous flow rate	(6.42 ± 0.72) μl min^−1^

Of relevance to our study is the work by Brown *et al.* [12], who obtained *in vivo* measurements of blood flow in various parts of the murine eye including the retina from birth to postnatal day (P) 16. Table 2 presents some of their findings. A clear trend of increase in retinal blood flow after P3 is observed. The authors attribute the large variability of the results (note standard deviation in table 2) to the natural variation in the time course of the remodelling processes involved.

**Table 2.** Retinal peak velocities measured in CD-1 mice by Brown *et al.* [12]. σ, standard deviation; *N*, number of samples.

age	velocity (cm s^−1^)	*σ* (cm s^−1^)	*N*
P0	0.32	0.09	5
P1	0.71	0.29	7
P2	1.22	0.29	6
P3	0.58	0.07	6
P4	4.62	1.09	5
P5	2.70	0.94	5
P6	5.26	1.79	5
P7	4.30	0.07	6
P8	3.53	1.73	7
P10	2.79	0.14	5
P12	4.35	0.52	5

Several authors have also studied the typical pressure difference driving flow in the retina, the so-called ocular perfusion pressure (OPP). The pressure at the central retinal artery is often approximated with mean arterial pressure (MAP) measurements at eye level (e.g. carotid arterial pressure [25] and subclavian artery [26]). The effective venous pressure is considered equivalent to the intraocular pressure (IOP) [27]. Table 3 summarizes the values of MAP and IOP reported by several authors. OPP values of approximately 57 mmHg are consistently reported across species. Finally, retinal blood flow is known to be autoregulated by the modulation of retinal vessel compliance in response to MAP and IOP variations [13,26].

**Table 3.** Values of mean arterial pressure (MAP) and intraocular pressure (IOP) reported in the literature for different species.

authors	species	MAP (mmHg)	IOP (mmHg)
Wright & Harris [25]	16-week-old mice	68.2 ± 2.0	11.6 ± 0.4
Hardy *et al.* [26]	1–3-day-old piglets	70 ± 6	13 ± 2

From a rheological point of view, blood is a shear-thinning fluid (i.e. its viscosity is a decreasing function of shear rate [28]). When flowing at sufficiently large shear rates (typically greater than approx. 1000 s^−1^) blood can be modelled as a Newtonian fluid (i.e. constant viscosity) with no significant effect on the simulated haemodynamics (see Bernabeu *et al.* [29] and references therein). However, at lower shear rates, viscosity quickly increases due to, for example, red blood cell (RBC) aggregation. Our work concerns simulation of blood flood in small arterioles, venules and capillaries where shear rate is expected to be lower than the aforementioned threshold. In fact, Nagaoka & Yoshida [30] measured shear rates as low as (606 ± 115) s^−1^ in the venules of the human retina. Therefore, we will take the shear-thinning properties of blood into account in order to improve the fidelity of the shear stress computed in our model. Table 4 compiles some of the values of murine blood viscosity as a function of shear rate available in the literature. Other rheological properties derived from the presence of RBCs, such as the Fåhræus–Lindqvist effect (e.g. [33]), will not be considered.

**Table 4.** Blood viscosity as function of shear rate in mice.

authors	animals	shear rate (s^−1^)	viscosity (mPa s)
Vogel *et al.* [31]	4–7-month-old C57Bl/6 mice	2	18.94 (average, *n* = 11)
		5	13.33
		11	10.52
		23	8.07
		45	6.31
		90	5.96
		225	4.91
		450	3.85
Windberger *et al.* [32]	4–8-month-old BALB/c mice	0.7	13.36 (median, *n* = 37)
		2.4	10.56
		94	4.87

Finally, we note that experimental data on haemorheology changes during development is limited. Windberger *et al.* [34] observed a steady increase in blood viscosity in rabbits and cats from fetal stages to adulthood. The changes were more pronounced within lower shear rates regimes: from 3.00 to 9.29 mPa s at 0.7 s^−1^ and from 2.48 to 3.62 mPa s at 94 s^−1^ during the first 30 days of life in rabbits. Owing to the scarcity of available data, we will not include this effect in our model.

The experimental data on pressure distributions and haemorheological properties surveyed in this section will be used to set up our flow simulations. This is done due to the impossibility of obtaining such information directly from our experimental model. The data summarized in table 1 will be used to validate our experimental measurements of vessel diameter and *in silico* estimates of blood velocity and flow rate. We now turn our attention to previously proposed models of retinal blood flow.

### 2.3. Previous modelling and simulation studies

In one of the earliest works on retinal haemodynamics modelling and simulation, Ganesan *et al.* [35, p. 1567] state that ‘although a relatively good understanding of the retinal anatomy and vascular network has been developed through extensive studies […] there is a complete lack of numerical modeling of retinal circulation in the literature’. In the same study, an image-based network model of the retinal vasculature was developed. The location and length of non-capillary vessel segments was extracted from confocal microscopy images of flat-mounted mouse retinas and a rule-based network model used to approximate the structure of the capillary bed. The haemodynamics were greatly simplified by considering vessel segments to be straight with piecewise constant radius and flow to be laminar (i.e. a one-dimensional network model), therefore neglecting complex fluid patterns that may appear in curved vessels even at low Reynolds numbers [36]. Their results show that WSS in the capillaries stays mainly in the 4–11 Pa range with values as high as 20 Pa. These magnitudes are substantially higher and with a much greater spread than those reported in the main retinal arteries and veins. Prior to this work, Liu *et al.* [37] also developed an image-based retinal flow model for the study of oxygen transportation in the retina. In this case, only a subset of the retinal vasculature (i.e. an artery and a number of branching arterioles) was reconstructed from a healthy human fundus camera image. The two-dimensional steady-state Navier–Stokes equations were solved in the domain. The model was used to predict pressure drops and oxygen saturation distribution.

More recently, Chen *et al.* [38] developed a mathematical model of blood flow in the zebrafish larvae midbrain vasculature (another typical model for the study of vascular development). The morphology of the vessel network was recovered from *in vivo* images at different developmental stages. Haemodynamics were also modelled using a laminar flow in straight circular pipe simplification. Both steady and pulsatile flow were compared with little difference in overall dynamics. The flow model was in turn coupled to a phenomenological model of changes in vessel diameter as a function of shear stress (without any explicit mechanism of endothelial cell migration or apoptosis). The resulting coupled model was used to predict vessel pruning in several zebrafish larvae midbrain vasculature samples with a reported 75% accuracy. These results support the hypothesis that WSS is a major factor in vessel pruning during angiogenesis.

Finally, Watson *et al.* [39] developed a comprehensive model of murine retinal angiogenesis including cell migration during sprouting, blood flow, oxygen distribution and the main chemotactic gradients involved in vessel development and pruning. In their work, vascular pruning was mainly driven by the downregulation of growth factors but no explicit mechanobiological mechanisms were considered. Blood flow simulation was also performed based on the one-dimensional network simplification described above.

The articles cited in this section demonstrate increasing interest in the modelling and simulation of retinal haemodynamics. Computational models have been developed for human, mouse and zebrafish retinal vasculature. A common application is the study of vascular development dynamics (angiogenesis in particular). In our opinion, the previous works, although seminal, share one or more of the following limitations: (i) the choice of imaging modality only allows the recovery of a subset of the retinal vasculature, (ii) flow dynamics are greatly simplified by the use of one- or two-dimensional approximations and (iii) the computer code developed is, to the best of our knowledge, not freely available. In this work, we aim at developing an open-source computational workflow for the generation of high-resolution estimates of the haemodynamic forces experienced by murine retinal vasculature based on confocal microscope images. The following section describes the methodology employed.

## 3. Material and methods

### 3.1. Image processing and three-dimensional model reconstruction

The preparation of retinal vascular plexus samples for imaging and analysis has been previously described in Franco *et al.* [40]. Briefly, plexus samples were collected from five- and six-day-old wild-type mouse pups and fixed with 2% paraformaldehyde in phosphate-buffered saline (PBS) for 5 h at 4°C, thereafter retinas were dissected in PBS. Blocking/permeabilization was performed using Claudio's blocking buffer (CBB), consisting of 1% FBS (Gibco), 3% BSA (Sigma), 0.5% triton X100 (Sigma), 0.01% Na deoxycholate (Sigma), 0.02% Na Azide (Sigma) in PBS pH = 7.4 for 2–4 h at 4°C on a rocking platform. Samples were stained with endothelial luminal marker (ICAM2) and incubated at the desired concentration in 1 : 1 CBB : PBS at 4°C overnight in a rocking platform. Finally, retinas were mounted on slides using Vectashield mounting medium (Vector Labs, H-1000) and imaged with a Carl Zeiss LSM780 scanning confocal microscope (Zeiss). Manual preprocessing of the plexus image was performed with Photoshop CS5 (Adobe) in order to remove major imaging artefacts, allowing a simple thresholding method to be used to produce a binary image of the entire retinal plexus segment. The binary image was skeletonized using a MATLAB (The MathWorks, Inc.) interface (http://www.mathworks.co.uk/matlabcentral/fileexchange/27543-skeletonization-using-voronoi) to the Voronoi tessellation algorithm implemented in the QHull library [41]. The radius at each Voronoi vertex was calculated from the maximum inscribed circle in two dimensions.

Based on the image skeleton and computed radii, a three-dimensional triangulation of the plexus luminal surface was generated by assuming vessel circular cross section with the VTK [42] and VMTK [43] libraries. This simplification is made based on our own histological analysis and on the observations of Feke *et al.* [15], who cite histological evidence of retinal arteries being circular in cross section, while retinal veins exhibit a higher tendency towards flattening. The volume contained within the surface was discretized as a regular grid in order to generate the computational domain necessary for simulation (see §3.2 for details). All the scripts developed are freely available at https://github.com/UCL/BernabeuInterface2014.

### 3.2. Simulation set-up

Let Ω be a three-dimensional domain with boundary 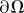. The CFD package HemeLB [8] was used to solve numerically the Navier–Stokes equations for generalized Newtonian incompressible fluids. For 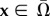 and time 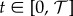
3.1
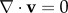
and3.2
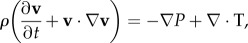
where *ρ* is the density, **v**(**x**, *t*) is the velocity vector, *P*(**x**, *t*) is the pressure, T(**x**, *t*) is the deviatoric part of the stress tensor3.3
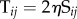
and3.4
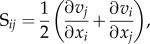
and *η* is the dynamic viscosity which may depend on the shear rate 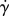, i.e. 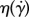,3.5
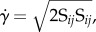
where *i*,*j* = 1, 2, 3 and summation over repeated indices is assumed. Note that in the case of Newtonian fluids, 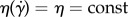.

Let 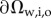 be the wall, inlet and outlet portions of the domain boundary, respectively, such that 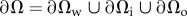. Equations (3.1)–(3.2) are closed with the following initial condition3.6
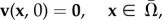
and boundary conditions3.7
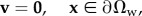
3.8
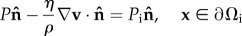
3.9
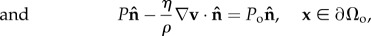
(i.e. a pressure drop problem as formulated by Heywood *et al.* [44] and Formaggia *et al.* [45]) where 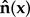, 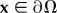, is the boundary normal vector and *P*_i,o_(*t*) are the pressures at the inlet and outlet, respectively. HemeLB uses the lattice-Boltzmann (LB) algorithm (see appendix A for a brief introduction) and runs efficiently on large scale high-performance computing resources [46]. HemeLB's source code is available under LGPL licence and can be downloaded from http://ccs.chem.ucl.ac.uk/hemelb. Simulations were run either locally (§4.1) or on up to 5040 cores of ARCHER, UK National Supercomputing Service (§4.3, appendices B and C).

**Figure 2.**
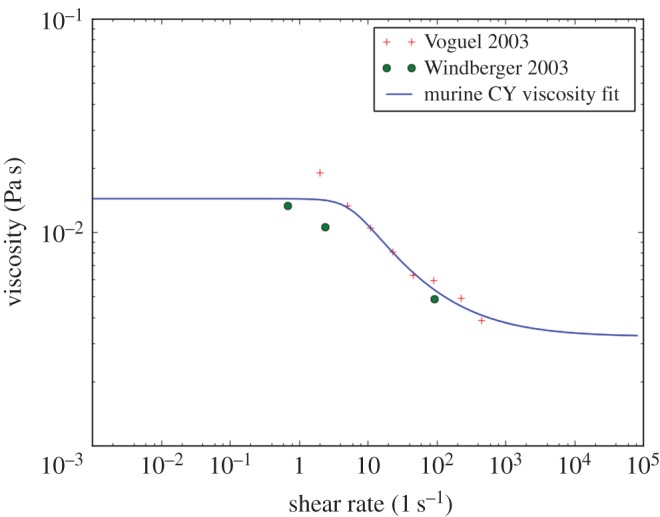
Reported values of murine blood viscosity for different shear rates and Carreau-Yasuda (CY) model fit. (Online version in colour.)

Experimental measurements were used to derive a functional form for 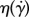 based on the Carreau-Yasuda (CY) mathematical model (e.g. [47])3.10
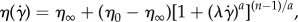
where *a*, *n* and *λ* are empirically determined to fit a curve between regions of constant *η*_∞_ and *η*_0_. This model defines three rheological regimes: a Newtonian region of viscosity *η*_0_ for low shear rate, followed by a shear-thinning region where *η* decreases with 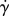; finally, a second Newtonian region of viscosity *η*_∞_ is defined for high shear rates. Equation (3.10) was fitted to the data in table 4 with the least-squares algorithm implemented in the gnuplot graphing utility (Gnuplot v. 4.6.3, http://www.gnuplot.info) giving the following results: *η*_0_ = 14.49 mPa s, *η*_∞_ = 3.265 mPa s, *λ* = 0.1839 s, *a* = 2.707, *n* = 0.4136. Figure 2 plots the data fit.

One of the many challenges when simulating blood flow in open domains—such as the subset of retinal vasculature that we present in figure 5—is the impact of the choice of inlet/outlet boundary conditions on the simulated haemodynamics. Ideally, one would use experimental measurements of flow rate and/or pressure in order to close the system. We could not obtain these data experimentally and relied on the data surveyed in §2.2. Pressures at the inlet and outlet were set to the values measured by Wright & Harris [25] and presented in table 3: 68.2 and 11.6 mmHg, corresponding to the mean pressure of the central retinal artery and vein, respectively.

The LB algorithm admits a number of different implementations of the no-slip boundary condition at the walls (see e.g. Lätt & Chopard [48] and Nash *et al.* [49] for surveys). We choose the method proposed by Bouzidi *et al.* [50] based on previous validation work [49]. In this work, we perform further validation as described in §3.3. Finally, we initialize the domain to a uniform density fluid at rest. The implications of this choice are discussed in §4.2.

### 3.3. Code verification methodology

In this study, we are interested in using HemeLB to simulate blood flow in a network of vessels of variable diameter, with differences of up to one order of magnitude (table 1). This is a challenging scenario because we must ensure that the spatial discretization is fine enough to capture all the features in the capillaries and resolve flow accurately, while keeping the problem computationally tractable due to the large number of fluid sites arising from the discretization of larger vessels. Furthermore, we are particularly interested in generating accurate estimates of WSS in vessels that are typically not aligned with the Cartesian grid, which can be challenging for regular grid based methods. For example, Stahl *et al.* [51] measured shear stress errors of up to 35% in the vicinity of the wall for non-lattice aligned channel flow with the LB algorithm and the so-called bounce-back implementation of the no-slip boundary condition [52].

In this section parameters are denoted with a tilde when given in lattice units and without when given in physical units. The LB time-step Δ*t* is used as a conversion factor for time and the voxel size Δ*x* for space such that e.g. diameter 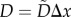. We also introduce the LB relaxation parameter (which controls the viscosity in the lattice, for more details refer to Chen & Doolen [53])3.11
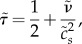
where 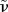 is the kinematic viscosity in lattice units3.12
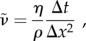
and 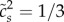 in the version of LB employed.

In the absence of experimental flow measurements to compare against our computer simulations, we propose setting up a set of benchmark simulations that capture the main flow and domain characteristics and compare the results against known analytical solutions. We will restrict ourselves to the simulation of steady, Newtonian and laminar flow in non-lattice aligned cylinders of diameter 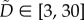 and length 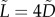. The orientation of the cylinder 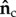 is chosen pseudorandomly from the unit sphere, subject to the constraint that 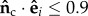, 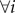. The value is3.13
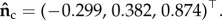
The laminar flow assumption is based on the Reynolds numbers reported in the literature for microcirculation (e.g. *Re* = 0.2, 0.05 and 0.0003 for arterioles, venules and capillaries, respectively [54]). We choose *Re* = 1 in our validation. The steady and Newtonian flow assumptions are made to simplify the analytical solution of the benchmarks considered. Their implications are discussed in §4.1. Finally, a parabolic velocity profile with maximum velocity,3.14
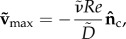
is imposed at the inlet [55].

The purpose of our validation study is twofold. First, to characterize the accuracy of the recovered haemodynamics as a function of the number of fluid sites across a given vessel. Second, to evaluate the accuracy of the computed WSS given our choice of implementation of the no-slip boundary condition [50], which has—to the best of our knowledge—not been done before.

In our first experiment, we compare the volumetric flow rate3.15
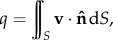
integrated over a lattice aligned cross-sectional plane (defined by point (0,0,0)^⊤^ and plane normal (0,0,1)^⊤^) with the analytical solution of Hagen–Poiseuille flow in an infinite cylinder3.16
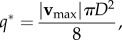
for a range of values of diameter 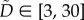 and relaxation time 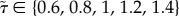.

In the second experiment, we compare T with the—appropriately rotated—analytical solution of the Hagen–Poiseuille shear stress tensor T′ in a cylinder of axis 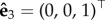 and radius *R* = *D*/2 assuming flow in the positive direction of the cylinder axis. For a given point in the domain **x** = (*x*_1_, *x*_2_, *x*_3_)^⊤^ such that 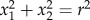, 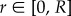, it can be shown that3.17
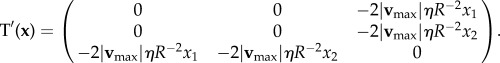


The tensor rotation is defined by the matrix 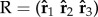,3.18
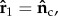
3.19
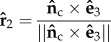
3.20
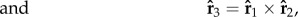
where 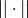 is the magnitude of a vector, such that3.21
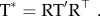


## 4. Results and discussion

### 4.1. Code verification

**Figure 3.**
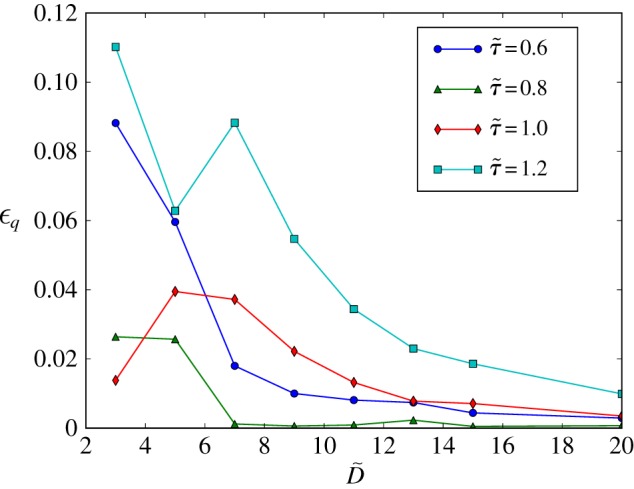
Hagen–Poiseuille flow in an inclined cylinder. Relative error on the computed flow rate as a function of vessel diameter 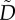 and lattice-Boltzmann (LB) relaxation time 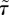. For 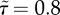, the total error is kept below 3% even for cylinders with just three lattice sites across. These results confirm the suitability of the LB algorithm for the simulation of flow in sparse geometries and porous media. The lines are a guide to the eye and bear no physical meaning. (Online version in colour.)

Figure 3 plots the relative error in the simulated flow rate4.1
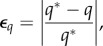
as a function of the cylinder diameter. We observe how the choice of 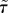 greatly affects the accuracy of the simulated haemodynamics. In agreement with similar analyses in the literature (e.g. [56]), the error is larger for values close to the stability threshold of 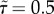 and for values greater than 1. A region of excellent accuracy is located around 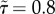. In that case, the relative error 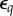 stays below 3% for all the values of 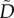 studied. The ability to simulate correct flow dynamics in channels with only a few lattice sites across has gained the LB algorithm wide acceptance for the simulation of flow in complex domains and porous media [57].

**Figure 4.**
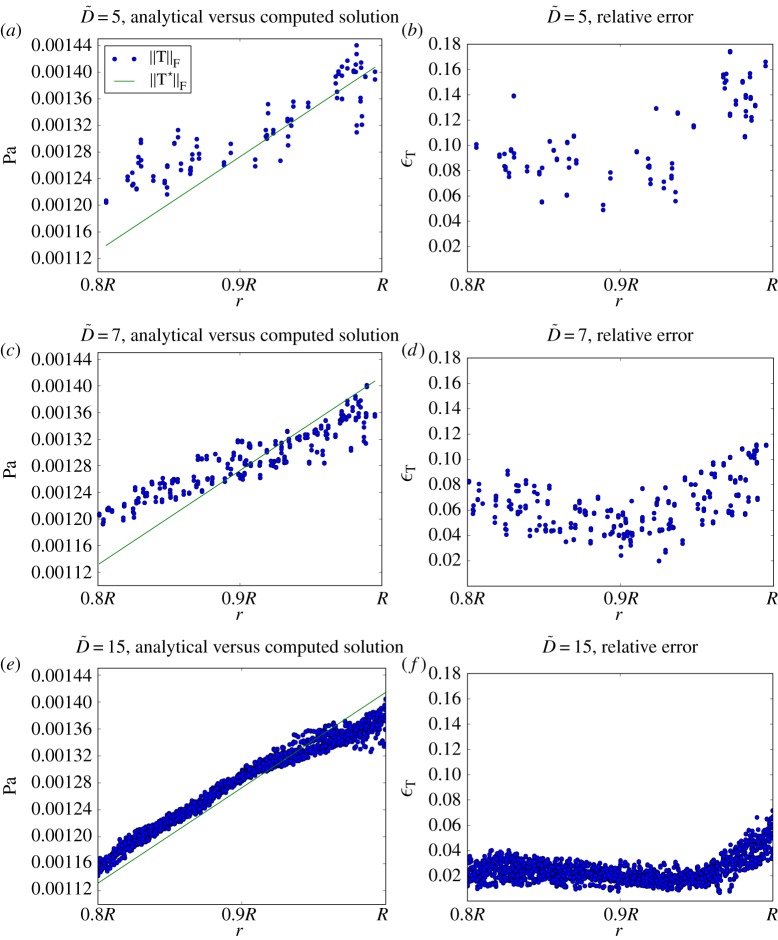
Hagen–Poiseuille shear stress in inclined cylinders of 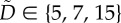. Norm of the analytical and computed stress tensors (panels 4*a*,*c*,*e*) and relative error between them (panels 4*b*,*d*,*f*). Results are presented for every lattice site with radius 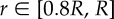. Agreement between computed and analytical solution improves with increasing 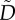. These results, with the Bouzidi *et al.* [50] implementation of the no-slip boundary condition, represent a substantial improvement over the 35% error reported by Stahl *et al.* [51] with the bounce-back method and 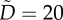. (Online version in colour.)

Figure 4 plots, for a range of values of 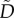, the computed and analytical solutions of the shear stress tensor (T and T^*^) as well as the associated relative error4.2
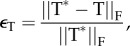
where 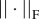 is the Frobenius norm of an *m* × *n* matrix 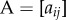
4.3
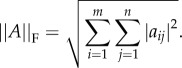
The choice of error norm in (4.2) ensures that error contributions given by individual components cannot compensate, hence being sensitive to rotation errors. We are mainly interested in the accuracy of the shear stress calculation in the vicinity of the vessel wall. Therefore, the results are presented for the subdomain defined by all the lattice sites with 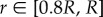.

It can be observed how the recovered shear stress follows the expected pattern of monotonic increase from zero at the cylinder axis (results not shown here) to its maximum value at the wall. However, a certain deviation exists compared to the analytical solution. For values of 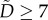, we observe a clear pattern of shear stress being overestimated in the [0.8*R*, 0.9*R*] region while being underestimated in [0.9*R*, *R*]. Figure 4*b*,*d*,*f* quantify this error. We observe how the largest error in the domain always occurs at the cylinder wall and that it decreases as 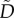 increases: from around 17% for 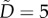 to around 7% for 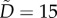. These results, with the Bouzidi *et al.* [50] implementation of the no-slip boundary condition, represent a substantial improvement over the 35% error reported by Stahl *et al.* [51] with the bounce-back method and 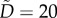, confirming the superiority of the former algorithm. More importantly, the WSS is consistently underestimated and the error decreases for 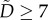. Therefore, one could implement an *a posteriori* correction of the computed shear stress tensor based on this knowledge. Such a development is beyond the scope of this paper and will be developed as part of a future study.

It is interesting to note that the errors in flow rate and shear stress are, to a large extent, decoupled. This is due to the characteristics of the LB algorithm as detailed in appendix A. In our work, we will define two rules for the accurate simulation of blood flow in our network of vessels of variable diameter. First, a minimum diameter of 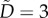 will be enforced throughout the domain. This will ensure that, for 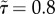, the general flow patterns produced are accurate. Second, values of shear stress in regions of interest will only be considered valid if 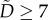. The error estimates reported in figure 3 will be taken into account in the presentation of our results.

Finally, we turn our attention to the implications of the use of a generalized Newtonian rheology model in our retinal flow simulations. In this section, we observed that 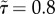 minimizes the error in the flow rate recovered. In the case of Newtonian fluids, regardless of the value of viscosity being simulated, one can choose 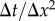 such that equation (3.11) yields the desired value of 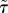 (this will obviously have an impact on computational cost). For generalized Newtonian fluids, 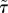 becomes a function of 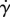 and will take values in the range 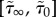. In the rheology model presented in §3.2, 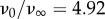, which would lead to impractical values of 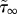 unless 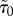 is chosen small enough. We will therefore choose the coefficient 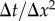 such that 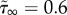 and 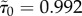. Based on the results in figure 3, this choice will yield an error of less than 4% in the flow rate recovered for 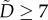.

### 4.2. Model reconstruction

**Figure 5.**
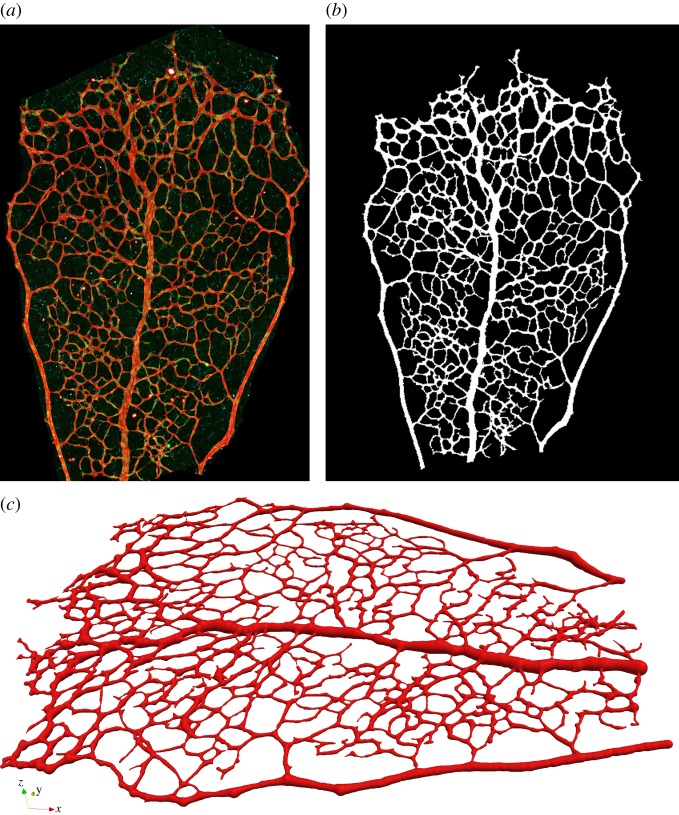
Subset of a wild-type P6 retinal plexus used to reconstruct one of our retinal blood flow models, namely P6A model. The original microscope image is segmented and the network skeleton and segment radii are computed. Based on these values, a three-dimensional volume is reconstructed assuming vessels of piecewise constant radius. (*a*) Original image. (*b*) Segmented image. (*c*) Reconstructed surface.

**Figure 6.**
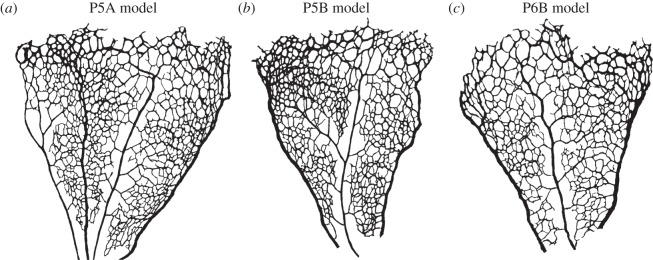
Binary masks defining the luminal surface of three retinal plexuses obtained at two different stages of development. All plexuses are presented with the area closer to the optic disc at the bottom of the image and the sprouting front at the top. In all samples studied, arteries tend to be thinner and have less daughter vessels than veins. Vessels close to the sprouting front tend to have less well-defined identity with luminal diameters comparable to arteries/veins. This is particularly notable in the P5 samples. Vessel density is also higher close to the sprouting front in P5 retinas.

The vascular plexus of wild-type retinas was stained with the luminal membrane marker ICAM2, and images were acquired using a confocal microscope as described in §3.1. Figure 5*a* shows a region of interest in one of the imaged retinas. It contains, on either side, two arterial segments coming from the optic disc and connecting with a segment of a retinal vein (centre of the image) through a dense capillary network. It can be appreciated how the network is more mature (e.g. vessel identity and branching patterns) closer to the optic disc (bottom of the image), while its structure is much more primitive and less remodelled in the periphery closer to the sprouting front (top of the image). Figure 5*b* presents the results of the image segmentation process. The algorithm described in §3.1 is used to first create a binary mask separating the luminal area and background tissue and second extract the network skeleton and radii. The latter are used to reconstruct the three-dimensional luminal surface under the assumption of vessel circular cross section (see §3.1 for a discussion). Figure 5*c* shows the reconstructed surface. We refer to this model as P6A. Figure 6 presents luminal surface binary masks for three additional P5 and P6 retinal plexuses. The same reconstruction algorithm is applied and the resulting models are referred to as P5A, P5B and P6B, respectively.

**Figure 7.**
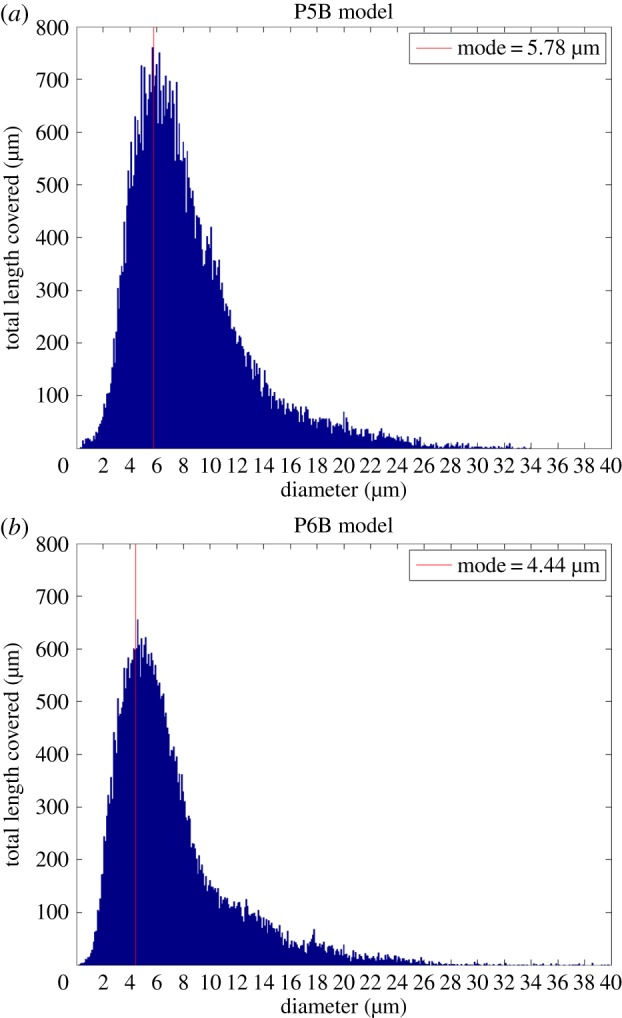
Network diameter histogram showing the aggregated total distance covered by vessels of a given diameter. Vertical lines indicate the mode of a lognormal probability distribution fit of each dataset. The values for the models not shown here are 5.51 μm (P5A) and 5.29 μm (P6A). We use these values as an estimate of the typical capillary diameter (the most common type of vessel in the network). Capillaries with diameter approaching 0 μm appear to be undergoing remodelling. Arterial and venular segments present higher diameters ranging up to 34 and 40 μm, respectively. (Online version in colour.)

Figure 7 plots a network diameter histogram (in terms of total distance covered by vessel segments of a given diameter) for models P5B and P6B. The largest diameter in the network are *D*_max_ = 34 and 40 μm, respectively, which occur along the retinal vein. The artery segments have diameters of up to 16 μm, with larger diameters closer to the optic disc. The bulk of the capillary bed has diameters approximately in the range 2–10 μm, with a reduced amount of vessels with smaller diameter. These results are substantially lower than the *in vivo* measurements presented in table 1. In addition, a small number of capillaries have diameters approaching 0 μm. To some extent, these discrepancies are expected as we are measuring the diameter of the internal luminal surface with extreme precision (unlike some of the works cited where only a generic measure of vessel calibre is given), including in our measurements vessel segments that appear to be undergoing regression (hence in the process of closing up). The diameter measured for the main arteries are, however, in better agreement with the *ex vivo* measurements obtained from corrosion casts by Ninomiya & Inomata [14]. Therefore, we cannot exclude that sample preparation and fixation protocols contribute to vessel shrinkage. Finally, we fit a lognormal probability distribution function to each histogram and use the distribution mode as an estimate of the typical capillary diameter (under the assumption that capillaries are the most common vessel type in the network). We observe a reduction in the typical capillary diameter between day 5 (5.51 and 5.78 μm) and 6 (4.44 and 5.29 μm). More experiments are required in order to assess the statistical relevance of these results but the implications of a systematic decrease in vessel diameter over time are important given that, for a constant flow rate, WSS is inversely proportional to the third power of the vessel radius. We plan to explore the relationship between changes in geometry and haemodynamics as part of a future study.

In order to ensure that 95% of the reconstructed network has 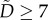, we choose the voxel sizes in table 5 for the discretization of each model. Appendix B presents a grid refinement study aimed at confirming that the choice of voxel size leads to spatially converged solutions.

**Table 5.** Voxel sizes employed in the discretization of the different flow models used in this work.

model	P5A	P5B	P6A	P6B
Δ*x*	0.5166 µm	0.5666 µm	0.5 µm	0.4166 µm

### 4.3. Simulations

Owing to its kinetic nature, the LB algorithm applied to steady flow problems in an initially quiescent domain requires the system to be advanced in time in order to overcome an initial transient. In order to monitor convergence, we evaluate the following convergence criterion at the end of each time-step *t*4.4
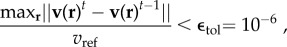
where 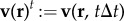 and *v*_ref_ is a velocity reference value chosen based on the data summarized in table 1, i.e. *v*_ref_ = 50 mm s^−1^. Only when this condition holds do we consider the simulation to have reached steady state. More efficient methods for LB initialization have been proposed (e.g. [58,59]) but we will not consider them in this work, because our approach remains computationally tractable.

**Figure 8.**
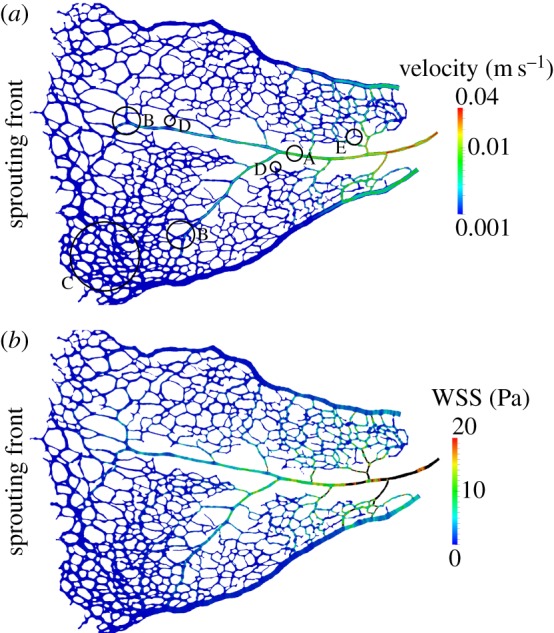
P5B simulation results: (*a*) velocity magnitude plotted on a cross section along the *z* = 0 plane. Velocity shows the expected parabolic profile across the vessel diameter. Velocity is higher in the artery located at the centre of the domain, in particular close to the optic disc. Velocity magnitude quickly decreases as the artery progresses towards the sprouting front and it stops being a preferential flow path at the points where its identity stops being clearly defined. (*b*) WSS magnitude plotted on the model surface. Areas of preferential flow tend to experience highest WSS magnitudes. WSS is generally low across the domain except for the arterial segment close to the optic disc and some first-order branches. WSS values higher than 20 Pa are considered unphysiological and the regions experiencing them are coloured in black. Black circles indicate regions of interest referenced in the manuscript.

Figure 8 presents results of a simulation with the P5B flow model and the inlet/outlet boundary conditions and rheological properties surveyed in §2.2. Velocity magnitude is plotted at the intersection of the model and the *z* = 0 plane (figure 8*a*). Our results show how velocities are larger in the central artery (see label A), in particular close to the optic disc. We also note that, as the artery progresses towards the sprouting front, the velocity magnitude decreases rapidly. Furthermore, it stops being a preferential flow path at the point where it meets areas of less well-established vessel identity close to the sprouting front (see e.g. B regions). Areas with undefined vessel identity are correlated with homogeneous velocity distributions (see e.g. C). There exists evidence of a considerable number of vessels having recently regressed along the path of the artery (see e.g. D branches) and the more developed first-order branches (see e.g. E). The two veins (top and bottom of the images) present fewer regressing profiles.

For a plane with normal 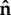, we define the traction vector4.5
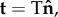
i.e. the force per unit area acting on that plane. Figure 8*b* plots traction magnitude 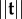 on the model surface (often referred to as WSS magnitude). We observe that areas of preferential flow correlate well with the areas experiencing larger WSS. By contrast, vessels in the sprouting front are under lower magnitudes of WSS. The model predicts values of WSS larger than 20 Pa, which can be deemed unphysiological based on the microvasculature WSS measurements reported in the literature: 14 Pa [54], approximately 20 Pa [35] or approximately 13 Pa computed from the values reported by Wright *et al.* [23] (under the assumption of Poiseuille flow). We believe that the WSS overestimation (mainly occurring at the central artery and some first-order branches) is due to the vessel shrinkage discussed earlier or other modelling errors.

**Figure 9.**
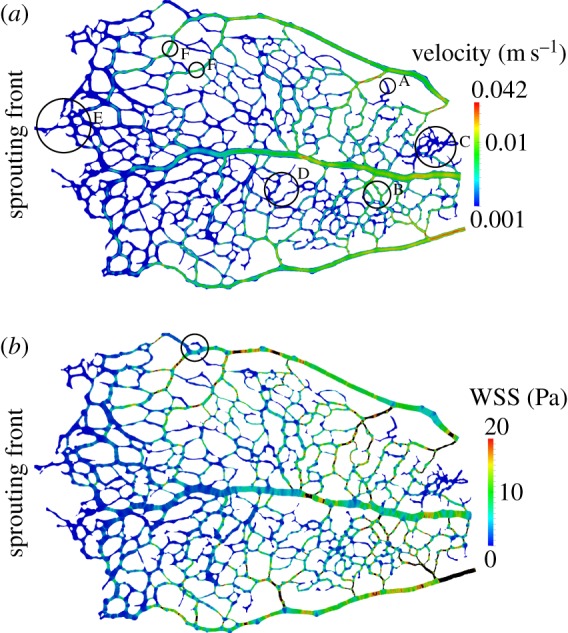
P6A simulation results: (*a*) velocity magnitude plotted on a cross section along the *z* = 0 plane. Velocity is higher in arteries, veins and segments directly branching from them close to the optic disc. Velocity magnitude is smaller in the sprouting front. However, vessels of preferential flow already exist in the sprouting front; potentially an early indicator of which vessels will survive the pruning process. (*b*) WSS magnitude plotted on the model surface. Areas of preferential flow tend to experience highest WSS magnitudes. WSS peaks are widely spread across the network. WSS magnitude tends to be lower at the junctions and many vessel segments present a high–low pattern due to local changes in vessel diameter. WSS values higher than 20 Pa are considered unphysiological and the regions experiencing them are coloured in black. Black circles indicate regions of interest referenced in the manuscript.

Figure 9 presents results of a simulation with the P6A flow model and the inlet/outlet boundary conditions and rheological properties surveyed in §2.2. Figure 9*a* plots velocity magnitude on the intersection of the model and the *z* = 0 plane. First of all, it can be appreciated how velocities are larger in arteries, veins and first-order vessels branching out from them. Highest peak velocities are around 42 mm s^−1^ (corresponding to mean velocities of 21 mm s^−1^ under Poiseuille flow assumption) and are in good agreement with the measurements by Wright and colleagues presented in table 1. Velocity distribution along a given vessel diameter displays the expected parabolic profile with zero velocity at the walls. Areas in more advanced state of pruning (typically closer to the optic disc, see region B) tend to present larger velocity magnitudes due to a reduction in vessel density. An exception to this trend is region C. In this case, we observe a region of very low flow (similar to the regions found in the less mature vascular plexus towards the periphery) in an area where pruning should be in a fairly advanced stage. Two explanations are possible: (i) that a recent vessel regression event has drastically reduced the total flow arriving to the area which in turn will trigger further vessel regression (similar to what can be observed in region A) or (ii) that a vessel segment connecting the area with the nearby artery was accidentally removed when preparing the sample. In contrast to the optic disc region, areas in the vicinity of the sprouting front experience lower velocity magnitudes (see region E). Nevertheless, even in this region, we can already appreciate segments of predominant flow (see e.g. F regions) rather than a totally homogeneous flow distribution. Taken together with observations from Chen *et al.* [38], we predict that these high-flow vessel segments are likely to survive the pruning process.

Figure 9*b* plots WSS on the model surface. We observe that areas of preferential flow correlate well with the areas experiencing larger WSS. However, in this case we do not see a decrease in WSS with increasing vessel order. WSS peaks are distributed throughout the domain in agreement with the observations by Ganesan *et al.* [35]. We also observe a complex distribution of WSS along individual vessel segments, with changes following local variation in vessel diameter.

**Figure 10.**
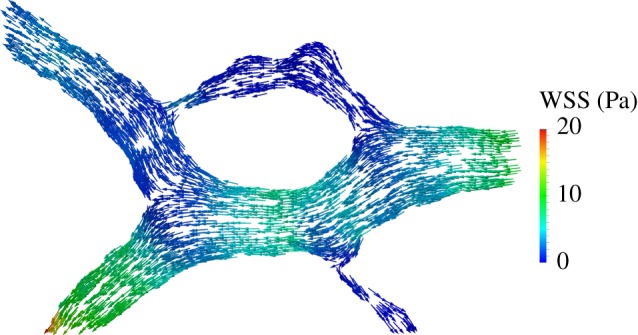
Traction vectors (of constant length and coloured according to magnitude) on the luminal surface of the region of interest highlighted in figure 9*b*. The loop branch undergoing regression (upper branch) experiences a much lower traction magnitude.

Figure 10 plots **t** on the surface of a subset of the domain (marked with a circle in figure 9*b*). Given the redundancy of a loop-like structure of this type and the distribution of diameters present, it can be assumed that the upper half of the loop is undergoing regression. This fact is in good agreement with the distribution of WSS, of much larger magnitude on the bottom section of the loop and vessel segments upstream and downstream from it.

In summary, the results presented in this section support the idea that vessel segments undergoing pruning tend to occur in regions of low flow (and hence low shear stress). We hypothesize that this process gradually reduces network density and as a consequence flow increases in the surviving vessel segments. This in turn prevents further pruning and contributes to vessel maturation.

### 4.4. Limitations of the study

The main limitations of this study are as follows. First, blood was modelled as a homogeneous fluid rather than a particle suspension. This is likely to have an impact on the WSS computed in small calibre capillaries. Xiong & Zhang [60] studied the changes in haemodynamics induced by the presence of RBCs flowing in a simplified model of a microvessel and found up to a 20% increase in the shear stress experienced by the luminal wall. Second, although blood was modelled as a shear-thinning fluid, other rheological properties such as the Fåhræus–Lindqvist effect (see §2.2) were not accounted for. Third, vessel cross section was assumed to be circular throughout the domain due to the lack of spatial information in the *z*-axis. As previously mentioned, there exists experimental evidence supporting this assumption in retinal arteries but not in veins [15]. This will have an impact on the haemodynamics recovered. Also, despite all our efforts when processing retina samples, we cannot be fully certain that no distortions in the vascular plexus were introduced. Next, due to the difficulty of measuring pressure or flow profiles at the model inlets/outlets *in vivo* and the absence of suitable data in the literature, only steady-state simulations were performed. We expect flow to be nearly in phase with pressure given the typical values of Womersley number (defined as the ratio between oscillatory inertial forces and viscous forces) encountered in retinal circulation (approx. 0.1 according to Liu *et al.* [37]). This makes us confident that flow has time to fully develop in each cardiac cycle and hence will be well approximated by an instantaneous pressure gradient. Nevertheless, there will still be substantial variations in WSS within any given cardiac cycle. Furthermore, the values of MAP and IOP used as inlet and outlet boundary conditions were obtained from adult animals. In appendix C, we perform a sensitivity analysis of these parameters. Finally, another source of variation in the predicted haemodynamics are the active and passive mechanical properties of retinal vessels. At the analysed stage, retinal arteries are already covered with a smooth muscle layer, which might contract/relax to control local flow (i.e. autoregulation) and therefore have an impact in flow patterns in downstream vessels.

## 5. Conclusion

In this work, we have presented a software pipeline for the creation of computational blood flow models based on confocal microscope images of the microvasculature. The pipeline has been applied to the development of flow models of the neonatal mouse retinal vasculature (a common animal model for the study of vascular development). The different software components used are released under open-source licences.

Using simplified benchmark problems, we have demonstrated the suitability of the lattice-Boltzmann (LB) algorithm for the simulation of blood flow in sparse and highly complex vascular networks. Our results indicate that a careful choice of the LB configuration parameters leads to accurate flow estimates in channels as narrow as three lattice sites across. Furthermore, we also showed that the implementation of the no-slip boundary condition proposed by Bouzidi *et al.* [50] produces acceptable estimates of WSS. We measured errors of approximately 10% and approximately 7% in channels 7 and 15 lattice sites wide, respectively. Being able to recover correct haemodynamics even at moderately coarse discretizations is fundamental to keep the problems under study computationally tractable.

In the study reported here, we investigated changes in haemodynamics during vascular remodelling. Blood flow models were generated from samples of retinal plexuses obtained at postnatal day (P) 5 and 6. Our simulations show that, in both cases, velocity and WSS are higher in arteries, veins and first-order capillaries closer to the optic disc. However, important differences in the distribution of velocity and WSS across the domain are observed when comparing both days (e.g. figures 8*b* and 9*b*). On the one hand, P5 simulations show a very homogeneous distribution of velocity and WSS across the capillary network with moderately high values only in the vicinity of the optic disc. On the other hand, simulations with the P6 flow model show a consistently higher and much more spatially complex distribution of velocity and WSS. Higher values are primarily located in regions in a more advanced state of remodelling (note, for example, the number of disconnected vessels undergoing regression). In the P6 case, branches of predominant flow can be also identified in the sprouting front.

We also analysed WSS in segments undergoing regression (e.g. figure 10) and observed vessel pruning occurring in regions of low shear stress. This process gradually reduces network density (through the removal of redundant segments) and is likely to lead to an increase in flow in the surviving vessel segments. We hypothesize that this will contribute to vessel maturation. Our results support the previously proposed modulation effect that haemodynamic forces have on developmental vascular remodelling [38].

The geometrical analysis of the vascular plexuses leads to two possible explanations for the increase in velocity and WSS observed between the P5 and P6 models. First, the increase may be a direct consequence of the observed decrease in typical capillary diameter (given the inverse relationship between WSS and the third power of the vessel radius and assuming that the total flow rate in the retina remains constant). Second, the progressive reduction in capillary bed density due to vessel regression may lead to an increase in flow (and hence WSS) in neighbouring vessels. We believe that both effects may play complementary roles in order to create the WSS gradients hypothesized to be behind vessel regression [38]. Further experiments are required in order to determine the relative importance of each of these effects and fully understand how they interact.

We are currently working on extending the modelling framework to include tissue mechanics and agent-based cellular modelling. Our goal is to develop an integrated computational framework for vascular mechanobiology research. In particular, we are interested in modelling the interplay between cellular molecular regulation and haemodynamic forces during vascular remodelling. Finally, the developed methodology should be applicable to other research domains where small vascular networks can be imaged but where experimental flow measurements are difficult to obtain.

## Figures and Tables

**Figure 11.**
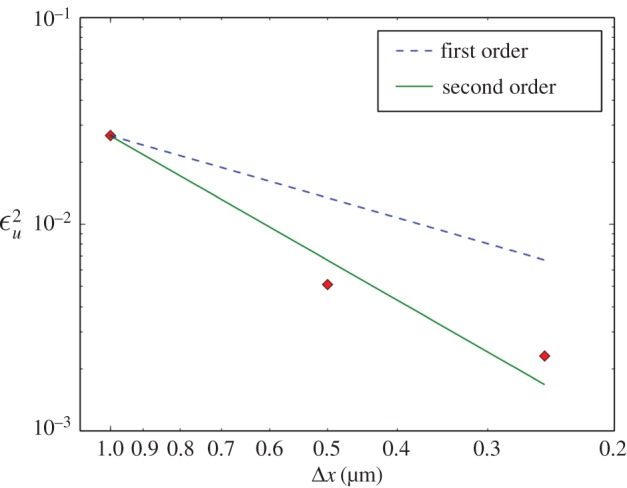
Velocity error residual 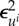 on simulations with the P6A flow model discretized with Δ*x^i^* = 1.0, 0.5 and 0.25 μm (diamond-shaped markers). In order to keep the analysis computationally tractable, 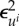 is computed with results obtained at the lattice sites located on the *z* = 0 plane only (this is the same subset of the results presented in figure 9). The lines are guides to the eye showing first-order (dashed) and second-order (solid) convergence. (Online version in colour.)

**Figure 12.**
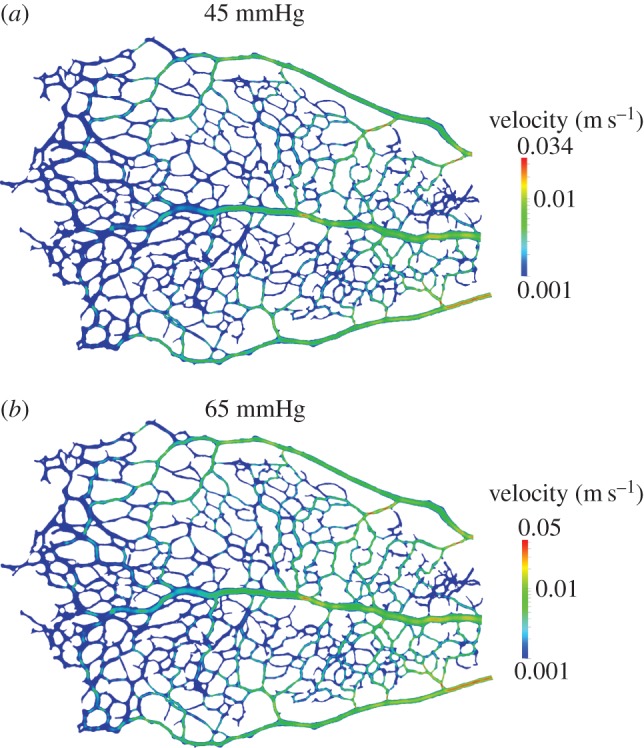
Simulation results: velocity magnitude plotted on a cross section along the *z* = 0 plane for OPP values of 45 and 65 mmHg. The logarithmic colour scales have been adjusted to range from 1 mm s^−1^ to the largest velocity in the domain. Branches of predominant flow and velocity gradients remain fairly constant despite moderate changes in OPP when compared with figure 9*a*.

## Appendix A. An introduction to the lattice-Boltzmann algorithm

In this section, we present a brief introduction to the lattice-Boltzmann (LB) algorithm. The interested reader can refer to well-cited references such as Chen & Doolen [53] and Aidun & Clausen [57] for a more in-depth presentation and analysis. LB operates at a mesoscopic level, simulating the evolution of a discrete-velocity approximation to the one-particle velocity distribution functions of the Boltzmann equation of kinetic theory, {*f_i_*(**r**, *t*)}. Computations are performed on a regular lattice discretization of , with grid spacing Δ*x*. The set of velocities {**c***_i_*} is chosen such that the distances travelled in one time-step (Δ*t*), **e***_i_* = **c***_i_*Δ*t*, are lattice vectors. When one only wishes to reproduce Navier–Stokes dynamics, the set is typically a subset of the Moore neighbourhood, including the rest vector. For three-dimensional simulations, the most commonly used sets have 15, 19 and 27 members. In this work, we employ the three-dimensional 15 velocity LB lattice (D3Q15).

Evolving the distribution functions in time involves two main steps. The first is known as the collision step, which relaxes the distributions towards a local equilibrium (the post-collisional distributions is often denoted as ):

A1

where  is a the collision operator. The second is known as the streaming step, where  are propagated along the lattice vectors to new locations in the lattice, defining the distribution functions at the next time step

A2

In this work, we employ the lattice Bhatnagar–Gross–Krook collision operator, which approximates the collision step as a relaxation process towards a local equilibrium,

A3

where *τ* is the relaxation time. This can be shown, through a Chapman–Enskog expansion (e.g. [61,53]), to reproduce the Navier–Stokes equations in the quasi-incompressible limit with errors proportional to the lattice Mach number squared. The kinematic viscosity *ν* is given by

A4

where

A5

is the speed of sound in the D3Q15 lattice. Equation (A 3) can be extended to simulate generalized Newtonian flows by locally varying *τ* according to (A 4) and an empirical characterization of the fluid viscosity (e.g. equation (3.10)).

For the equilibrium distribution, we use a second-order (in velocity space) approximation to a Maxwellian distribution

A6

where the weights *w_i_* and speed of sound *c*_s_ depend on the choice of velocity set. Other choices of  exist. Finally, the interested reader can refer to previous work by the authors (Nash *et al.* [49] and references therein) on the implementation of wall and open boundary conditions.

The macroscopic density *ρ*(**r**, *t*) and velocity **v**(**r**, *t*) at a fluid site can be calculated from the distribution functions by

A7

and

A8

The macroscopic pressure is related to the density by the ideal gas law

A9

A notable characteristic of the LB algorithm is that the shear rate tensor S can be computed at any lattice site from local information only. Let  be the non-equilibrium part of the distribution function. It can be shown that (e.g. [56])

A10

The main advantage of this approach is that S (and therefore , T and **t**) can be evaluated locally without the need of accessing simulation results generated at neighbouring lattice sites in order to approximate spatial derivatives of the velocity field. This makes parallel performance independent of the choice of rheology model or the need of computing WSS (a variable of primary importance in our work).

## Appendix B. Voxel size convergence analysis

In this section, we perform a grid refinement study of the simulations presented in §4.3. We want to assess whether the characterization of the discretization error done in §4.1 with a simplified domain and rheology model remains valid with more complex vessel networks and rheology models.

In order to confirm that the choice of Δ*x* in table 5 leads to spatially converged solutions, we generated a set of increasingly finer discretizations of the P6A flow model. The finest discretization that remained computationally tractable for us had . We used the results of this simulation as a reference solution and compared it against the results obtained with . Note that Δ*t* had to be modified according to (3.11) and (3.12) in order to keep  constant across all simulations (see §4.1 for more details). We define the error in the velocity field for a given discretization  as

B1

where *t*_conv_ corresponds, in each of the simulations, to the time where steady flow convergence has been achieved according to the stop criteria in equation (4.4) and  is the lattice site in the reference discretization closest to . Finally, we use the RMS of the error scaled by the predicted velocity range as our measure of error

B2

where *N* is the number of lattice sites in the  discretization or a subset of it.

Figure 11 plots the values of  computed on the subset of the P6A flow model presented in figure 9*a* (i.e. lattice sites on the *z* = 0 plane) for the choice of Δ*x* discussed above. The results show the LB configuration employed (i.e. lattice, collision operator and boundary condition implementation) displays second-order convergence behaviour. WSS shows similar convergence trends (results not presented here). These findings are in agreement with results previously published by the authors [49]. Finally, our grid refinement study shows how the choice of voxel sizes in table 5 leads to sufficiently spatially converged solutions. In the case of the P6A flow model, the results generated with Δ*x* = 0.5 µm have a relative error of only  when compared with the results obtained with finest discretization of the model that remained computationally tractable.

## Appendix C. Inlet/outlet configuration sensitivity analysis

In this section, we investigate the robustness of the results in §4.3 with regards to the choice of inlet and outlet boundary conditions. This is motivated by the fact that the choice of MAP and IOP in our flow model is based on data obtained from the literature (table 3) rather than directly measured from the animals used in the study. First, we want to investigate whether the general patterns of flow are affected by moderate changes in OPP (defined as the difference between MAP and IOP). Second, we want to quantify the relationship between OPP and blood velocity in the domain.

Figure 12 repeats the visualization in figure 9*a* for simulations with OPP 10 mmHg smaller and larger. The colour scale has been adjusted to range from 1 mm s^−1^ to the largest velocity in the domain in each case. It can be appreciated how the general patterns of flow and velocity gradients are greatly preserved from those in figure 9. As expected, the absolute values differ.

Table 6 presents the largest velocity magnitude recovered for a wider range of OPP values. It can be seen how peak velocity increases by approximately 7.6 mm s^−1^ for every 10 mmHg increase in OPP. The linear relationship between pressure difference driving flow in the domain and peak velocity indicates that flow occurs in the Stokes regime (i.e. inertial forces are small compared with viscous forces). This result is expected given the typical Reynolds numbers reported in the literature for microcirculation (e.g. *Re* = 0.2, 0.05 and 0.0003 for arterioles, venules and capillaries, respectively [54]).

**Table 6.** Peak velocity in the domain as a function of the ocular perfusion pressure (OPP).

OPP (mmHg)	domain peak velocity (mm s^−1^)
25	18.97
35	26.65
45	34.31
55	41.96
65	49.61

We conclude that the inlet/outlet boundary conditions play a secondary role in determining the flow patterns in the network, hence no modulation in the WSS gradients experienced by the endothelium is expected via changes in OPP (this does not include changes due to autoregulation of the vessels themselves). Changes in vessel network geometry are expected to be the main drivers behind haemodynamic reorganization during development. This observation supports the idea that regression of a given vessel segment will affect the flow patterns in nearby segments, leading to potential increases in WSS that may contribute to vessel maturation.
